# Robust antibody and cellular responses with an improved DNA vaccine alone

**DOI:** 10.1186/1742-4690-9-S2-P15

**Published:** 2012-09-13

**Authors:** N Hutnick, DJ Myles, K Muthumani, M Morrow, J Yan, AS Khan, NY Sardasai, DB Weiner

**Affiliations:** 1 University of Pennsylvania, Philadelphia, PA, USA; 2 Inovio Pharmaceuticals, Blue Bell, PA, USA

## Background

The recent results of the RV144 trial demonstrate that HIV-specific antibodies may provide protection from infection. DNA vaccines may represent an important HIV vaccine platform since they are safe, relatively inexpensive to manufacture, and stable at room temperature. However, the platform has been used largely as a prime modality limited to induce low level CD4 T cell responses in NHP and humans. In this study we sought to improve our current DNA vaccines to induce HIV-specific antibodies with plasmid vaccination alone.

## Methods

Groups of 5 Indian Rhesus Macaques were vaccinated with pHIV consensus gag, pol, and clade C envelopes delivered IM with in-vivo electroporation at weeks 0, 4 and 12. Immunogenicity was measured two weeks after each dose.

## Results

Three doses of an HIV DNA vaccine alone induced robust cellular and antibody responses. Despite using clade C based enveloped, high binding titers were detectable against gp120s from multiple clades. Neutralizing titers were in the 100’s range to a panel of clade B and C tier 1 viruses. These data establish that designed DNA envelop antigens can drive functional immunity in NHP.

## Conclusion

Multiple improvements to DNA vaccine technology have significantly enhanced the immunogenicity of the platform. Just three doses of a plasmid based HIV vaccine induced robust binding and neutralizing antibodies as well as effector T-cell responses in NHP. We are currently expanding the immunity induced by these constructs through novel DNA adjuvants as well as in prime-boost combinations.

